# Osteogenic Potential and Bioactive Profiles of *Piper sarmentosum* Ethanolic Extract-Treated Stem Cells

**DOI:** 10.3390/ph16050708

**Published:** 2023-05-07

**Authors:** Intan Zarina Zainol Abidin, Anis Nabilah Johari, Muhammad Dain Yazid, Zaidah Zainal Ariffin, Herryawan Ryadi Eziwar Dyari, Shahrul Hisham Zainal Ariffin

**Affiliations:** 1 Centre for Research and Graduate Studies, University of Cyberjaya, Cyberjaya 63000, Malaysia; 2 Department of Biological Science and Biotechnology, Faculty of Science and Technology, Universiti Kebangsaan Malaysia, Bangi 43600, Malaysia; 3 Centre for Tissue Engineering and Regenerative Medicine, Universiti Kebangsaan Malaysia Medical Centre, Cheras 56000, Malaysia; 4 Faculty of Applied Sciences, Universiti Teknologi MARA, Shah Alam 40450, Malaysia; 5 Department of Earth Sciences and Environmental, Faculty of Science and Technology, Universiti Kebangsaan Malaysia, Bangi 43600, Malaysia

**Keywords:** *Piper sarmentosum*, differentiation, osteoblast, human peripheral blood stem cells, volatile compounds

## Abstract

*Piper sarmentosum* is a well-known traditional herbal plant in various diseases treatments. Multiple scientific studies have also reported various biological activities exhibited by the plant’s extract, such as antimicrobial, anticarcinogenic and antihyperglycemic activities, and, in addition, a bone protective effect in ovariectomized rats has been reported. However, no known *Piper sarmentosum* extract is involved in osteoblast differentiation using stem cells. Our study aims to identify the potential of *P. sarmentosum* ethanolic extract to induce osteoblast differentiation of human peripheral blood stem cells. Prior to the assay, the proliferation ability of the cells was observed for 14 days and the presence of hematopoietic stem cells in the culture was determined by the expression of *SLAMF1* and *CD34* genes. During the differentiation assay, the cells were treated with *P. sarmentosum* ethanolic extract for 14 days. Osteoblast differentiation was examined using an (alkaline phosphatase) ALP assay, by monitoring the expression of osteogenic gene markers and by von Kossa staining. The untreated cells served as the negative control, while cells treated with 50 µg/mL ascorbic acid and 10 mM β-glycerophosphate acted as the positive control. Finally, the determination of the compound profile was performed using a gas chromatography-mass spectrometry (GC-MS) analysis. The isolated cells were able to proliferate for 14 days during the proliferation assay. The expression of hematopoietic stem cell markers was also upregulated during the 14 days assay. Following the differentiation induction, the ALP activity exhibited a significant increase (*p* < 0.05) from day 3 of the differentiation assay. A molecular analysis also showed that the osteogenic markers *ALP, RUNX2, OPN* and *OCN* were upregulated compared to the positive control. The presence of mineralized cells with a brownish-stained morphology was observed, indicating the mineralization process increased in a time-dependent manner regardless of the concentration used. There were 54 compounds observed in the GC-MS analysis, including β-asarones, carvacrol and phytol, which have been shown to possess osteoinductive capacities. Our results demonstrate that the ethanolic extract of *P. sarmentosum* can induce osteoblast differentiation of peripheral blood stem cells. The extract contains potent compounds which can potentially induce the differentiation of bone cells, i.e., osteoblasts.

## 1. Introduction

Natural products, specifically herbal medicinal plants, are gaining more interest from consumers for treating diseases. The number of commercialized derivatives from medicinal plants is increasing and they can be easily purchased. These natural medicines are deemed to have more advantages because numerous compounds that exist in a particular plant can work synergistically and exert a multitarget efficacy compared to conventional medicines that target one specific disease [1]. Natural products are also preferred due to their low cost, fewer side effects and suitability for long-term consumption [2]. *Bacopa monniera, Glycyrrhiza glabra* and *Polygonum odoratum* are a few examples of medicinal plants studied as treatments for Alzheimer’s disease, psoriasis and breast cancer, respectively [1].

*Piper sarmentosum*, known as ‘Kaduk’, is one of the most well-known herbs that locals use as an alternative medicine. *P. sarmentosum* belongs to the family *Piperaceae* and can be easily found in tropical and subtropical regions of the world [3]. This plant is traditionally used by locals to treat minor wounds and diseases such as diabetes, hypertension, coughs, rheumatism and asthma [4,5]. The high content of compounds in *P. sarmentosum* enables it to possess numerous properties such as antibacterial, antihypertensive, antihyperglycemic and antimalarial properties and tissue regeneration of embryos and caudal fins [6,7,8]. Previous in vivo studies have shown that *P. sarmentosum* extracts exhibited the ability to act as a bone protective agent by restoring the microstructure and bone mass in glucocorticoid-induced osteoporosis and in ovariectomized and adrenalectomized rats [9,10]. These health benefits are attributed to the synergistic effect of compounds abundantly found in the raw plant extract. Nevertheless, the involvement of compounds in bone remodeling should be identified to give us a better understanding of the mechanisms involved. However, the effect of plant extracts on a cellular level, specifically on human peripheral blood stem cell (hPBSC) differentiation into osteoblasts, which is the building block for bones, is still unknown.

Bone structures undergo a continuous remodeling process throughout life to provide the optimum function by maintaining the mechanical strength of the bone through calcium homeostasis [11]. Two important cells in bone tissue remodeling are osteoclast and osteoblast cells that degrade bone by dissolving the matrix protein and reform the bone by producing extracellular proteins. The imbalance in this process may result in bone diseases such as osteoporosis, which is a skeletal disease that is characterized by a low bone mass and a deteriorating microarchitecture of the bone tissue, which can lead to fragile bones and fractures [12]. Currently, few drugs are used as a treatment for osteoporosis, they include estrogen, calcitonin and bisphosphonates. Unfortunately, the administration of these drugs might lead to side effects such as an increased risk of uterus cancer, myalgias and osteonecrosis [13,14]. Hence, to reduce the dependency on such drugs, the role and potential of *P. sarmentosum* in bone remodeling should be determined. Although a few previous in vivo studies have shown the ability of *P. sarmentosum* to improve bone health, the effect of its extract in the bone remodeling process on the cellular level is still not well studied.

In this study, the osteoblast differentiation potential was observed using human peripheral blood stem cells (hPBSCs). This process enables hPBSCs to differentiate into a variety of matured cells such as osteoclasts, chondrocytes and osteoblasts, making it a good model for differentiation studies [15,16]. These findings serve as a good indicator of the potential of *P. sarmentosum* to be used as a complementary medicine to treat bone diseases by inducing bone formation.

The objective of this study was to identify the potential of *P. sarmantosum* ethanolic extract to induce osteoblast differentiation of human peripheral blood stem cells (hPBSC) and determine the compound profile. Prior to a differentiation assay, the proliferation ability of the cells was determined using trypan blue exclusion dye staining. Then, a reverse transcription-quantitative polymerase chain reaction (RT-qPCR) was carried out to observe the expression of hematopoietic stem cell markers (*CD34* and *SLAMF1*). Following this, the differentiation of osteoblasts was observed using three approaches which were biochemical (ALP assay), molecular (RT-qPCR) and morphological (von Kossa staining). The expressions of four osteoblasts gene markers were observed, i.e., *ALP, RUNX2, OPN* and *OCN*, during molecular observations. Finally, the compounds present in *P. sarmentosum* ethanolic extracts were screened using a GC-MS analysis.

## 2. Results

### 2.1. Proliferation Capacity and Expression of Hematopoietic Stem Cell Markers

**Figure 1:**
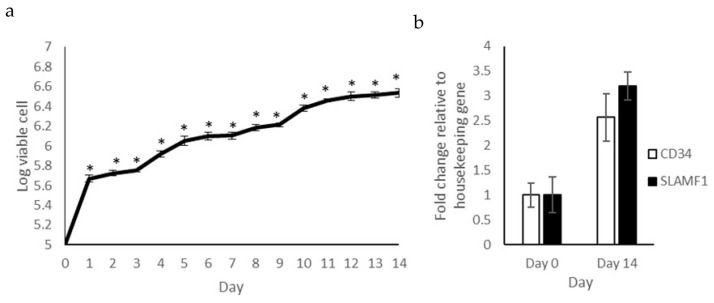
Hematopoietic stem cell proliferation potential and molecular stemness marker. (**a**) The proliferation of isolated mononucleated cells was measured every day for 14 days. (**b**) The expressions of *SLAMF1* and *CD34* were analyzed using RT-qPCR on days 0 and 14. The expressions of both genes were normalized to the housekeeping gene (*GAPDH*). The means ± standard error of the mean are given for three independent experiments (*n* = 3). * denotes significant differences (*p* < 0.05) compared to day 0.

The ability of self-renewal is one of the characteristics of a stem cell. The proliferation of isolated cells was observed before conducting the differentiation assay as shown in Figure 1a. The number of viable cells increased significantly (*p* < 0.05) starting from day 1 to day 14, exhibiting a approximately 34-fold increase on day 14 compared to day 0. This shows that the isolated cells can survive and proliferate well for 14 days in the provided in vitro culture environment.

Other than the ability for self-renewal, the expressions of hematopoietic stem cell markers *SLAMF1* and *CD34* were observed using the RT-qPCR technique. To observe the regulation of gene expression, RNA was extracted on day 0 and day 14, as shown in Figure 1b. The expressions of both markers were significantly upregulated (*p* < 0.05) on day 14 compared to day 0. *SLAMF1* exhibited a 3.2-fold increase while *CD34* exhibited a 2.6-fold increase. The high proliferation capacity of the in vitro culture and the increases in stemness markers indicate the presence of stem cells in the isolated cell population.

### 2.2. Viability and ALP-Specific Activity during the Differentiation Assay

**Figure 2:**
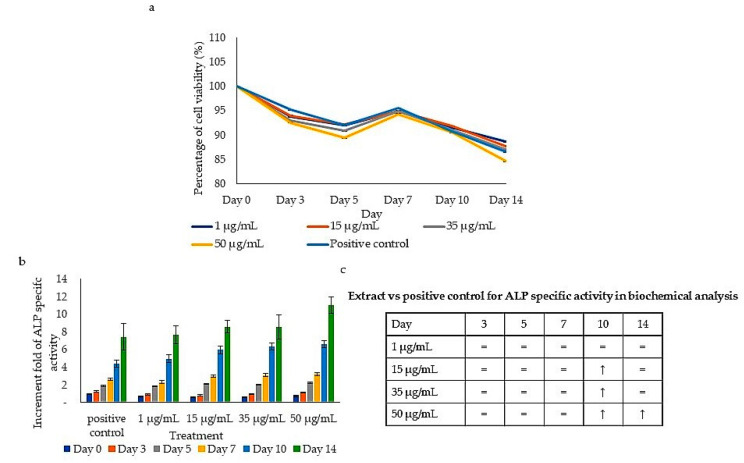
Viability and ALP profiles during differentiation. (**a**) The percentage of cell viability of the treated cells on days 0, 3, 5, 7, 10 and 14 normalized to the untreated cells (untreated cells act as 100%). (**b**) The increase in ALP-specific activity after treatment with a range of concentrations of extract for 14 days. (**c**) A comparison of ALP-specific activity in extract-treated cells to the positive control. Means ± standard error of the mean are given for three separate experiments (*n* = 3). ↑: significantly higher expression (*p* < 0.05) and =: similar expression (*p* ≥ 0.05) compared to the positive control.

The viability of the treated cells was observed during differentiation. Figure 2a shows the percentage of cell viability of human PBSCs on days 0, 3, 5, 7, 10 and 14 of the differentiation assay. Overall, cells treated with 50 µg/mL exhibited the lowest cell increase starting from day 3 to day 14 onwards when compared to the negative control. At day 14, the lowest cell viability was 84.6% (50 µg/mL), followed by 35 µg/mL (87.1%), 15 µg/mL (87.8%) and 1 µg/mL (88.6%) (Figure 2a).

ALP-specific activity is often used as an osteogenesis marker [17,18,19,20]. A gradual increase in ALP-specific activity was shown after treatment with all concentrations (1, 15, 35 and 50 µg/mL) starting from day 3 in a time-dependent manner (Figure 2b). Treatment with 50 µg/mL exhibited the highest ALP-specific activity after 14 days with a 9.69-fold increase, followed by 35 µg/mL (8.86-fold increase), 15 µg/mL (8.68-fold increase) and 1 µg/mL (8.27-fold increase) when compared to the negative control. In addition, the ALP-specific activities of cells treated with all concentrations of extract on day 14 were 8 to 10 times higher than on day 0. Our study showed that the increases in ALP-specific activity at high treatment concentrations (15, 35 and 50 µg/mL) were significantly higher (*p* < 0.05) on day 10 and on day 14 when compared to the positive control on the respective days (Figure 2c). Meanwhile, the ALP-specific activity throughout treatment with a lower concentration (1 µg/mL) was similar to the positive control (*p* ≥ 0.05). This indicates that *P. sarmentosum* extract induced an increase in the ALP activity when compared to the negative control and significantly induced ALP activity at a later stage (days 10 and 14) at higher concentrations when compared to the positive control.

### 2.3. Expression Profiles of Osteoblast Markers

**Figure 3:**
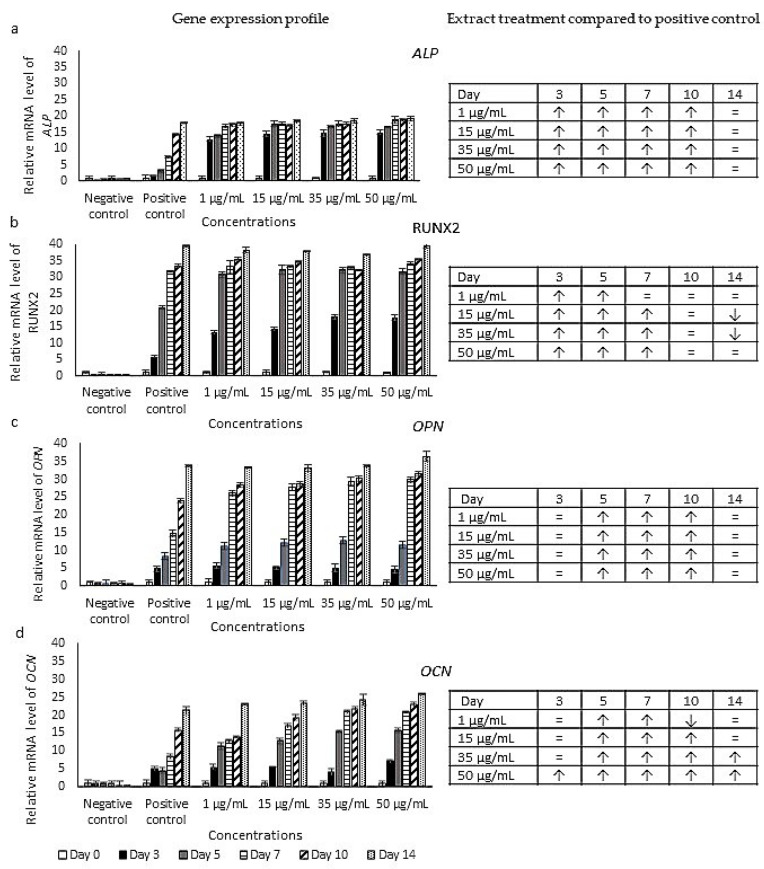
Osteoblast gene profiles during treatment, i.e., (**a**) *ALP*, (**b**) *RUNX2*, (**c**) *OPN* and (**d**) *OCN*. The expression of osteoblast gene markers corresponds to the comparisons of the expressions in the extract-treated cells to the positive control. Means ± standard error of the mean are given for three separate experiments (*n* = 3). ↑: significantly higher expression compared to the positive control (*p* < 0.05), ↓: Significantly lower expression compared to the positive control (*p* < 0.05), =: similar expression compared to the positive control (*p* ≥ 0.05).

A molecular analysis of the osteoblast marker, i.e., *ALP*, *RUNX2*, *OPN* and *OCN*, expressions was carried out using the RT-qPCR technique (Figure 3). The expression of the *ALP* gene in the extract-treated cells shows an increase in a time-dependent manner. A significant increase was observed on days 3, 5, 7 and 10 for all treatment concentrations when compared to the positive control. The gene was expressed 12 to 14 times higher on day 3 of the treatment (1–50 µg/mL) while only 1.8 times higher in the positive control culture (Figure 3a). The expression of the *RUNX2* gene in the extract-treated cells was significantly upregulated on days 3, 5 and 7 compared to the positive control. On the other hand, significant decreases were exhibited after treatment with 15 and 35 µg/mL on day 14 of the differentiation assay (Figure 3b). The *OPN* and *OCN* genes were also upregulated throughout the 14-day treatment, with the highest expression on day 14 compared to the negative control. The highest expressions of the *OPN* gene exhibited a 33–37-fold increase, while *OCN* gene expressions exhibited a 23–25-fold increase. Figure 3c shows a significant increase (*p* < 0.05) in *OPN* gene expression in treatments at all concentrations on days 5, 7 and 10 when compared to the positive control. Meanwhile, cells treated with the highest concentration (50 µg/mL) exhibited a significant increase in *OCN* gene expression (*p* < 0.05) in all periods of treatment (days 3, 5, 7, 10 and 14) as compared to the positive control (Figure 3d). Treatment with 1–35 µg/mL exhibited similar expressions during early treatment (day 3) and increased significantly from day 5 to day 10. In addition, an increase was still noted on day 14 after treatment with 35 µg/mL, while similar expressions were exhibited on day 14 after treatments with 1 and 15 µg/mL when compared to the positive control. A significant decrease was only shown after 1 µg/mL treatment on day 10 of the differentiation assay. These results show that the expressions of *ALP* and *RUNX2* increased significantly in early treatment, starting on day 3, and became similar or decreased significantly on day 14. Meanwhile, significantly higher expressions of *OPN* and *OCN* were recorded in the later stages of treatment, starting on day 5, with the exception of treatment with 50 µg/mL of *P. sarmentosum* extract, which was able to significantly increase the expression of *OCN* from day 3.

### 2.4. Morphology of Mineralized Cells

**Figure 4:**
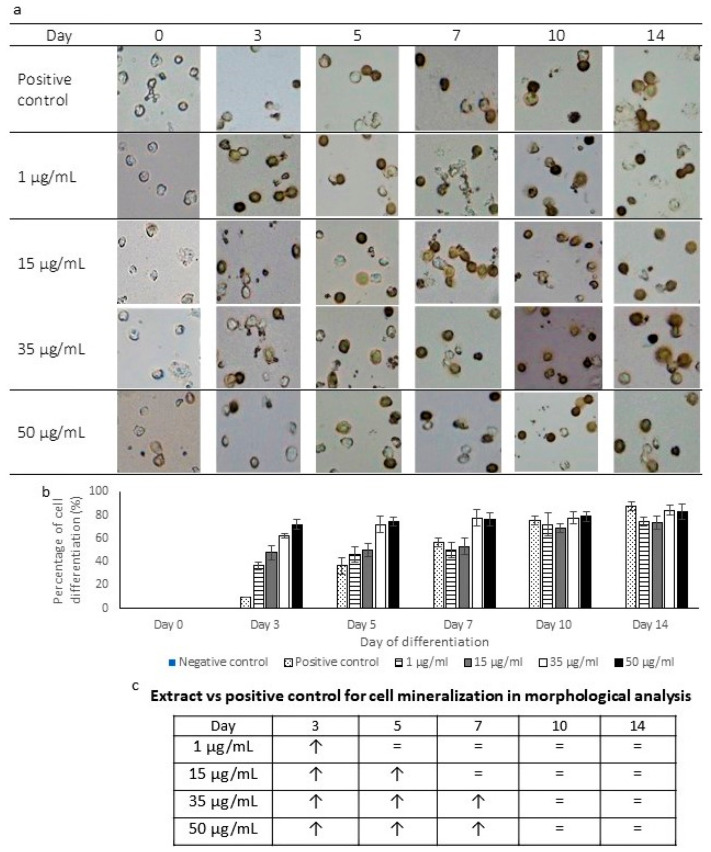
Mineralized hematopoietic stem cells during treatment. (**a**) Representative images of mineralized cells following von Kossa staining (magnification ×400) (**b**) The percentage of mineralized cells on days 0, 3, 5, 7, 10 and 14. (**c**) A comparison of cell mineralization in extract-treated cells with the positive control. Means ± standard error of the mean are given for three separate experiments (*n* = 3). ↑: significantly higher expression compared to positive control (*p* < 0.05), =: similar expression compared to positive control (*p* ≥ 0.05).

The mineralization rate of osteoblasts was observed using von Kossa staining. Figure 4a shows the morphology of mineralized cells after being treated with *P. sarmentosum* ethanolic extracts for 14 days. On day 0 of treatment, all von Kossa stained cells appeared unstained and translucent when observed under a microscope. A positive mineralization morphology was shown after day 3 of treatment onwards. Cells that proliferated were stained and appeared darker (brown or black), as observed in Figure 4a. The differentiated cells could be observed to increase proportionally to the period of treatment at all four concentrations.

The numbers of both differentiated and undifferentiated cells were counted to determine the percentage of differentiation that occurred (Figure 4b). On day 0, there was no cell differentiation in all treatments. Then, a significant increase (*p* < 0.05) in the cell differentiation percentage was observed in 1 µg/mL (day 3), 15 µg/mL (days 3 and 5), 35 µg/mL and 50 µg/mL (days 3, 5 and 7) treatments when compared to the positive control (Figure 4c). The highest percentage of differentiation was recorded on day 14 of the positive control culture (87%), followed by the treatment using 35 µg/mL (84%), 50 µg/mL (83%) and 1 µg/mL (81%). On the other hand, there were no stained cells present in the negative control culture. This shows that the extract induced higher cell mineralization at the early phase (day 3) and still induced mineralization until day 7 at higher extract concentrations compared to the positive control. The mineralization exhibited by the extract-treated cells increased proportionally to the concentrations of the extract.

### 2.5. Compound Content in P. sarmentosum Ethanolic Extracts

GC-MS analysis is a technique to identify volatile compounds present in a plant extract. Hence, this analysis was carried out to reveal the compounds in *P. sarmentosum* ethanolic leaf extracts. The GC-MS analysis revealed 54 volatile compounds in the ethanolic extracts of *P. sarmentosum* leaves. 1-dodecane took the shortest time to be eluted (9.972 min), while 3,4-dimethoxycinnamic acid was the last compound to be analyzed at 33.736 min. The most prevalent compounds were 2,4-di-tert-butylphenol (32.24%), phenol, 2,5-bis (1, 1-dimethylethyl) (32.24%), γ asarone (10.97%), benzene, 1,2,3-trimethoxy-5-(2-propenyl) (10.97%) and asarone (10.31%).

**Figure 5:**
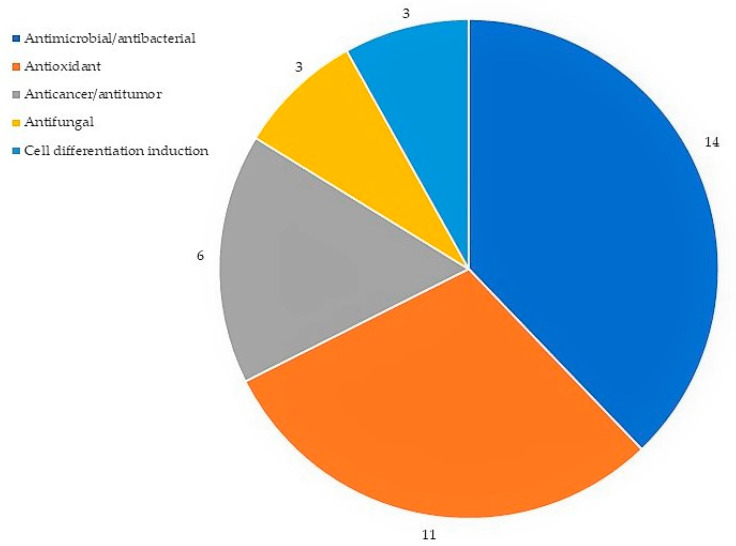
Frequencies of biological activities of volatile compounds in *P. sarmentosum*.

Out of these 54 volatiles compounds, 21, or approximately 39%, have been reported to exhibit biological activities, including antimicrobial, antioxidant, anticancer and antifungal properties and cell differentiation induction (Table 1). In addition, 10 out of 21 comp ounds have been reported to possess more than one biological activity. The most reported biological activities associated with the compounds screened are antimicrobial and antioxidant properties (Figure 5). Furthermore, the GC-MS analysis identified the presence of three volatile compounds that have been reported to be involved in cell differentiation induction activity, which are phytol, β-asarone and carvacrol. Another important biological activity that is potentially involved in bone health is antioxidant activity, which also has been reported with the second highest frequency (Figure 5).

## 3. Discussion

### 3.1. Stem Cell Characterization and Osteoblast Differentiation Induction

The ability to renew itself for an indefinite period is a characteristic of a stem cell. The proliferation ability of the isolated cells exhibited that human PBSCs were present in the culture before the differentiation process. Other than proliferation activity, a molecular approach was also taken by analyzing the expression of two hematopoietic stem cell markers, i.e., *SLAMF1* and *CD34. SLAMF1* is categorized under the SLAM subfamily, which are involved in the regulation of the proliferation and activation of lymphocytes. *SLAMF1* is also important for maintaining the undifferentiated state of hematopoietic stem cells by stimulating the proliferation process [42,43], explaining the upregulation during the proliferation assay (Figure 1a). *CD34* is well known to be expressed in human hematopoietic stem cells. The expression of this gene is involved in cell cycle entry, mobilization and metabolic activation [44,45].

Cell differentiation consists of four stages: proliferation, extracellular matrix (ECM) production, ECM maturation and apoptosis [46]. The reduction in the cell proliferation percentage after treatment with the extract, Figure 2a, indicates that the cells have entered the differentiation phase. This occurs because of the inability of the cells to carry out both proliferation and differentiation at one time [17,47]. ALP, which is involved in the release of phosphate for mineralization, has been used as a marker to indicate osteoblast differentiation. The upregulation of ALP activity is one of the main events that occur during the early phase of osteogenesis, thus it is commonly used by researchers to determine osteoblast formation [17,48]. Hydrolyzation by ALP provides the chemical conditions that eventually lead to mineral deposition, which can be visualized using von Kossa staining [49]. The increase in the ALP profile from day 3 to day 14 observed in the present study signifies the regulatory potential of *P. sarmentosum* extracts on ALP-specific activity that leads to the osteoblast differentiation process.

In this study, the increases in ALP-specific activity and differentiated cell percentage were also supported by the expression of osteoblasts-specific markers using RT-qPCR, i.e., *ALP*, *RUNX2*, *OCN* and *OPN*. These markers have commonly been used in other osteoblast differentiation studies to represent the molecular observation of the mineralization process [50]. *ALP* is a known marker for osteogenic differentiation and is secreted to promote the mineralization of the extracellular matrix. The upregulation in *ALP* expression and the increase in ALP activity indicate the potential of *P. sarmentosum* ethanolic extract to induce calcification during osteoblast development. Then, the expression of *RUNX2* results in higher differentiation and mineralization of osteoblasts because of its role in the early stage of the differentiation process [50]. *RUNX2* is also involved in inducing the expression of *OCN,* which is a mature osteoblast marker that is expressed during the late phase of osteoblast formation [51]. The significantly higher expression of these early genes, *ALP* and *RUNX2*, on days 3, 5, 7 and 10 shows that the extract is highly capable of initiating the differentiation process. OCN is the most synthesized protein by osteoblasts, making it one of the most important markers during bone mineralization [52]. Another osteoblast gene marker observed was *OPN*, which is a non-collagenous bone matrix protein that is expressed during the late phase of the mineralization process. During in vitro differentiation, the secretion of OCN and OPN will lead to the final osteoblast phenotypic marker, which is the mineralization of bone nodules as visualized during von Kossa staining [53]. Meanwhile, OPN is involved in the bone formation process via osteoblasts which enable the attachment of osteoclasts [54]. The expressions of *OPN* and *OCN* were significantly higher than the positive control starting from day 5, indicating the maturation phase experienced by the extract-treated cells. However, a high concentration of *P. sarmentosum* extract (50 µg/mL) was observed to increase the expression of *OCN* throughout the experiment, while the concentration of the extract did not affect the expression of *OPN*. Our study shows that *P. sarmentosum* ethanolic extract promotes the expression of these osteoblast markers, showing the ability of the extract to induce the formation of mineralized nodules in the treated cells similar to the positive control (50 µg/mL ascorbic acid + 10 mM β-glycerophosphate).

von Kossa staining is a common method to visualize the free inorganic phosphate present, wherein the silver ions in the stain react with phosphates and carbonates in calcium deposits, producing black precipitates [55], as observed in Figure 4a. This staining method has been carried out to observe the osteoblast differentiation of various cells such as dental pulp stem cells, human exfoliated deciduous teeth and MC3T3-E1 cell lines [20,48,56]. Our study also reported similar results to these studies by exhibiting an increased number of brownish to black cells as the mineralization process occurs continuously following the differentiation induction by *P. sarmentosum* extract. The effects of *P. sarmentosum* ethanolic extract on ALP-specific activity and the visualized mineralization process were comparable to the use of ascorbic acid and β-glycerophosphate, suggesting the ethanolic extract of *P. sarmentosum* could serve as a good inducer of osteoblasts differentiation.

### 3.2. Compound and Biological Activity of Piper sarmentosum Ethanolic Extract

The results of the GC-MS analysis unveiled the presence of 54 compounds in the ethanolic extracts of *P. sarmentosum* leaves. Among these 54 compounds, 21 of them have been reported to exhibit biological activities. The most prevailing compounds identified with the highest peak areas were 2,4-di-tert-butylphenol, phenol, 2,5-bis (1, 1-dimethylethyl), γ asarone, benzene, 1,2,3-trimethoxy-5-(2-propenyl) and asarone. Most of these compounds have been reported to exhibit biological activities. 2,4-di-tert-butylphenol is a lipophilic phenol that can be found in numerous organisms such as bacteria, fungi and plants [55,56,57]. This phenol exhibits multiple bioactivities such as antioxidant, anti-inflammatory, cytotoxicity and antifungal activities [24,57,58]. These compounds might be important in osteoblast differentiation induction shown in our study as phenolic compounds have exhibited a positive effect on bone homeostasis [59]. On the other hand, multiple studies also have reported a wide range of bioactivities exhibited by β-asarone and γ-asarone, such as antioxidant, anticancer, anti-ischemia and insecticidal properties [26,34,60,61]. Following these major compounds, 1-tetradecene and 1-pentadecene (peaks of 2.77% and 4.65%) have been reported to exhibit antimicrobial and antioxidant activities, respectively [23,62]. On top of that, three compounds have been reported to be involved in cell differentiation induction activity. The compounds mentioned are β-asarone, phytol and carvacrol, which were involved in the differentiation of neuron cells, osteoblast and endothelial cells, respectively [33,39,40]. Although there are only three compounds that are associated with cell differentiation activity, the presence of the other compounds in the extract is worth taking note of, as there are studies that have reported that the biological activities of plant-derived products are contributed to by the synergistic effect between compounds [63]. Hence, the list of compounds in this study can be used as a reference for further studies to identify the potential components that might also be present in different osteoinductive extracts. Our GC-MS analysis also showed that *P. sarmentosum* ethanolic extract contains several potential highly volatile compounds or specifically antioxidants that are beneficial for the bone remodeling process. The presence of antioxidant compounds is crucial in maintaining the bone remodeling process as the reactive oxygen species (ROS) can induce apoptosis of osteoblasts that will favor osteoclastogenesis. Imbalanced and increased osteoclastogenesis causes a high turnover of bone remodeling and bone loss [64]. Hence, antioxidants present in *P. sarmentosum* could prevent over-formation of osteoclasts and promote bone formation.

## 4. Material and Methods

### 4.1. Preparation of Plant Material

The leaves of *P. sarmentosum* were collected from the Forest Research Institute of Malaysia (FRIM), Kuala Lumpur, Malaysia (GPS coordinate: 3°14′7.80″ N 101°38′9.59″ E), and identified by a plant taxonomist with a voucher specimen FRI 45870. The collected *P. sarmentosum* leaves were cleaned and dried in an oven at 50 °C. The dried leaves were ground to a fine powder for extraction. Approximately 20 g of dry powdered leaves was extracted using 200 mL of ethanol in Soxhlet apparatus for six hours, followed by condensation of the extract in a rotary evaporator by evaporating the ethanol. Finally, the condensed extract was left in the fume hood for seven days to ensure a complete removal of the solvent. The fully dried extract was kept at −20 °C in a sticky form until further use. Before further analyses, the extract was diluted with 1% dimethyl sulfoxide (DMSO) to generate a range of stock concentrations, i.e., 10 µg/mL to 500 µg/mL.

### 4.2. Collection, Isolation and Culture of Human Peripheral Blood Stem Cells

The osteoinductive effect of *P. sarmentosum* was tested on human peripheral blood stem cells. Prior to sample collection, donor consent and ethics approval (reference number: UKM PPI/111/8/JEP-2019-612) were obtained from the Research Ethics Committee Universiti Kebangsaan Malaysia (RECUKM). Three peripheral blood samples were collected from healthy adults aged between 18 and 25 years old. Then, the collected blood was diluted with Hanks Balanced salt solution in a ratio of 1:3 before being layered onto Ficoll-Paque^TM^ PLUS (1:1.5) for density gradient centrifugation. The blood was then centrifuged for 20 min at 400× *g* at 27 °C. The second layer containing mononucleated cells was removed and washed three times using phosphate-buffered saline (PBS). The pelleted cells were resuspended and cultured in a 24-well plate in a complete medium consisting of α-medium essential (AMEM), 2% (*v*/*v*) penicillin-streptomycin and 10% (*v*/*v*) new-born calf serum (NBCS). These cells were cultured in an incubator at a temperature of 37 °C in the presence of 5% CO_2_ for 7 days before further experiments [16,17,18].

### 4.3. Proliferation Ability of the Isolated Cells

Cells were seeded at a density of 1 × 10^5^ cells/mL and cultured in the proliferation medium for 14 days. The viability and proliferation capacity of the isolated cells were observed every day for 14 days using trypan blue exclusion dye. The unstained and stained cells were observed under a microscope and viable cells were determined using a hemacytometer [17].

### 4.4. Molecular Characteristics of Hematopoietic Stem Cells

Total RNA was extracted from the treated cells using Trisure reagent (Bioline, Meridian Bioscience, Memphis, TN, USA) according to the manufacturer’s instructions. One milligram of total RNA was used in reverse transcription to produce complementary DNA using a Sensifast cDNA Synthesis Kit (Bioline, Meridian Bioscience, Memphis, TN, USA). A real-time polymerase chain reaction was later performed using a Thunderbird SYBR qPCR Mix kit (Toyobo, Osaka, Japan) with the following cycling conditions: 95 °C for 30 s at the holding stage, then followed by a two–step amplification (40 cycles of denaturation at 95 °C for 5 s and extension at the respective temperatures as listed in Table 2 for 10 s). The primer sequences for hematopoietic stem cell markers (*SLAMF1* and *CD34*) and extension temperatures are listed in Table 2.

### 4.5. Induction of Osteoblast Differentiation Using P. sarmentosum Ethanolic Extract

The cells were seeded at 1 × 10^5^ cells/mL for an osteoblast differentiation assay [18]. The prepared *P. sarmentosum* ethanolic extracts were added into the culture at final concentrations from 1 µg/mL to 50 µg/mL. Cells treated with a cocktail consisting of 50 µg/mL ascorbic acid and 10 mM/L β-glycerophosphate served as the positive control, while untreated cells in the complete medium were the negative control. The differentiation of the cells was observed on days 0, 3, 5, 7, 10 and 14.

### 4.6. Cell Viability of Differentiated Cells

The viability and proliferation ability of the treated cells were observed for 14 days using the trypan blue exclusion dye approach [17]. On days 0, 3, 5, 7, 10 and 14, the cells were harvested and stained with trypan blue in a ratio of 1:1 and the cells were counted using a hemacytometer under the microscope. The cell viability was normalized to the untreated cells on the respective days and presented as a percentage.

### 4.7. Alkaline Phosphatase (ALP) Assay

PBS was used to wash 1 × 10^5^ cells/mL and the cells were lysed using 0.1% Triton. The total protein content was determined using Bradford reagent for 5 min at room temperature and measured at 595 nm. For the ALP assay, the cells were incubated in 0.1 mol/L sodium bicarbonate–sodium carbonate buffer, 2 mmol/L magnesium sulfate and 6 mmol/L p-nitrophenyl inorganic phosphate for 30 min at 37 °C. The reaction was stopped by adding 1 mol/L sodium hydroxide and the absorbance was measured at 405 nm. The ALP activity was represented as specific activity, which was determined by the unit activity per total protein (mg). One unit of ALP activity represents the hydrolysis of 1 µM p-nitrophenol per min at 37 °C. The ALP-specific activity was presented as a percentage, which was normalized to the negative control [17].

### 4.8. Molecular Characteristics of Osteoblast Cells

Total RNA was extracted from the treated cells using Trisure reagent (Bioline) and used in reverse transcription to produce complementary DNA using a Sensifast cDNA Synthesis Kit. A real-time polymerase chain reaction using a Thunderbird SYBR qPCR Mix kit was later performed. The primer sequences and extension temperatures for osteoblast markers (*ALP, RUNX2, OPN* and *OCN*) are listed in Table 1.

### 4.9. von Kossa Staining

Approximately 1 × 10^5^ cells/mL were washed using PBS and fixed onto a glass slide using 10% (*v*/*v*) formalin in PBS for 30 min. Then, the slide was rinsed using deionized water and the cells were stained with a 5% (*v*/*v*) silver nitrate solution. After 30 min, the slide was rinsed and 5% (*v*/*v*) sodium carbonate in 25% (*v*/*v*) formalin was added for 5 min followed by 5% (*v*/*v*) sodium thiosulfate for 2 min. Finally, the slide was rinsed and left to air dry before being observed under the microscope. A mineralized cell was defined as a cell stained dark brown or black [17].

### 4.10. Gas Chromatography-Mass Spectrometry (GC-MS) and Compound Identification

For GC-MS analyses, the sample was placed in a 30 m × 0.25 mm ID × 0.25 μm capillary column. The injection port temperature was set at 290 °C in splitless mode and helium (99.999%) was used with a flow rate of 36.3 cm/s. The sample was diluted in DMSO and injected using an AOC-20i + s autoinjector. The temperature was programmed as follows: 5 min at 50 °C, heated at 2 °C/min to 300 °C and held for 10 min. The mass spectral scan range was set at 30–700 (*m*/*z*). The compounds present were compared to the database of The National Institute of Standards and Technology (NIST).

### 4.11. Statistical Analysis

Our data were analyzed using a two-way ANOVA followed by Dunnet’s post hoc test. A result was considered to be statistically significant at a *p* value of <0.05.

## 5. Conclusions

The potential for osteogenesis induction was determined by the increase in specific activity of the ALP enzyme, the expression profiles of osteoblast markers and the mineralization of differentiated cells. A concentration of 50 µg/mL of *P. sarmentosum* ethanolic extract is suggested to possess the highest osteoblast-differentiation-inducing properties. Following the differentiation assay, screening of volatile compounds using GC-MS also indicated that *P. sarmentosum* contains 54 compounds, with three (β-asarone, carvacrol and phytol) compounds already reported to have cell differentiation induction potentials, 19 with other biological activities and 33 that are still not well studied. This array of compounds may contribute individually or synergistically to the ability to induce the differentiation of osteoblasts. Hence, further studies are required to investigate the presence of the other compounds that are responsible for the osteoinductive capacity exhibited in this study. In conclusion, *P. sarmentosum* ethanolic extracts could potentially replace the available osteoblast differentiation factor for bone formation.

## Figures and Tables

**Table 1:** Screening of compounds using a GC-MS analysis.

No	Retention Time (RT)	Peak Area (%)	Name of the Volatile Compound	Biological Activity
1	9.972	2.63	1-dodecane	Antimicrobial, antioxidant and antifungal [21]
2	10.101	0.3	1-dodecanol	Antibacterial [22]
3	13.885	2.77	1-tetradecene	Antioxidants [23]
4	15.91	32.24	2,4-di-tert-butylphenol	Antioxidant, antimicrobial and anticancer [24]
5	15.91	32.24	Phenol, 2,5-bis (1,1-dimethylethyl)	Anticancer [25]
6	16.885	10.97	γ-asarone	Anticancer, antimicrobial [26]
7	16.885	10.97	Benzene, 1,2,3-trimethoxy-5-(2-propenyl)	Antimicrobial [27]
8	17.099	6.23	2-tetradecene, (E)	Antioxidant [28]
9	17.099	6.23	Cetene	Antioxidant [29]
10	17.23	4.65	1-pentadecene	Antioxidant, antimicrobial [23]
11	18.026	0.55	Copaene	Antioxidant [30]
12	18.159	1.66	1,3-benzodioxole, 4,5-dimethoxy-6-(2-propenyl)	Antifungal [31]
13	18.53	10.31	Asarone	Antioxidant [32]
14	18.71	5.16	β-asarone	Cell differentiation inducing [33], antioxidant, antimicrobial, anticancer and antifungal [34]
15	19.918	1.99	E-15-heptadecenal	Antioxidant, antimicrobial [35]
16	19.918	1.99	1-octadecene	Anticancer, antimicrobial and antioxidant [36]
17	19.918	1.99	5-octadecene, (E)	Antimicrobial [37]
18	20.55	0.96	Neophytadiene	Antimicrobial [38]
19	26.146	0.16	Phytol	Cell differentiation inducing, antimicrobial [39]
20	30.995	0.5	Carvacrol	Cell differentiation inducing, antimicrobial [40]
21	33.736	1.31	3,4-dimethoxycinnamic acid	Antitumor, antimicrobial [41]

**Table 2:** Primer sequences used for hematopoietic stem cell characterization and osteoblast detection.

Gene	Sequences (5′–3′)	Extension Temperature (°C)
*GAPDH*	Forward- GACCACTTTGTCAAGCTCATTTC	60
	Reverse- CTCTCTTCCTCTTGTGCTCTTG	
*SLAMF1*	Forward- GGAAAGCAGGAAGGAGGA	60
	Reverse- GCAGCCCAGTATCAAGGT	
*CD34*	Forward- TAGCCAAGTCTGCCAACTATTC	55
	Reverse- CCAACATACCACCCTCCATTT	
*ALP*	Forward-GGAGTATGAGAGTGACGAGAAAG	54
	Reverse- GAAGTGGGAGTGCTTGTATCT	
*RUNX2*	Forward- CGGAATGCCTCTGCTGTTAT	55
	Reverse- TGTGAAGACGGTTATGGTCAAG	
*OPN*	Forward- GCTAAACCCTGACCCATCTC	56
	Reverse- ATAACTGTCCTTCCCACGGC	
*OCN*	Forward- CCTGAAAGCCGATGTGGT	57
	Reverse- GGCAGCGAGGTAGTGAAGA	

## Data Availability

Data is contained within the article.
